# Targeting the DNA Damage Response Machinery for Lung Cancer Treatment

**DOI:** 10.3390/ph15121475

**Published:** 2022-11-27

**Authors:** Katharigatta N. Venugopala

**Affiliations:** 1 Department of Pharmaceutical Sciences, College of Clinical Pharmacy, King Faisal University, Al-Ahsa 31982, Saudi Arabia; kvenugopala@kfu.edu.sa; 2 Department of Biotechnology and Food Science, Faculty of Applied Sciences, Durban University of Technology, Durban 4000, South Africa

**Keywords:** DDR, homologous recombination (HR), NHEJ, BER, NER, ATMi, DNA-PKi, PARPi

## Abstract

Lung cancer is considered the most commonly diagnosed cancer and one of the leading causes of death globally. Despite the responses from small-cell lung cancer (SCLC) and non-small cell lung cancer (NSCLC) patients to conventional chemo- and radiotherapies, the current outcomes are not satisfactory. Recently, novel advances in DNA sequencing technologies have started to take off which have provided promising tools for studying different tumors for systematic mutation discovery. To date, a limited number of DDR inhibition trials have been conducted for the treatment of SCLC and NSCLC patients. However, strategies to test different DDR inhibitor combinations or to target multiple pathways are yet to be explored. With the various biomarkers that have either been recently discovered or are the subject of ongoing investigations, it is hoped that future trials would be designed to allow for studying targeted treatments in a biomarker-enriched population, which is defensible for the improvement of prognosis for SCLC and NSCLC patients. This review article sheds light on the different DNA repair pathways and some of the inhibitors targeting the proteins involved in the DNA damage response (DDR) machinery, such as ataxia telangiectasia and Rad3-related protein (ATR), DNA-dependent protein kinase (DNA-PK), and poly-ADP-ribose polymerase (PARP). In addition, the current status of DDR inhibitors in clinical settings and future perspectives are discussed.

## 1. Introduction

Lung cancer is considered one of the commonest malignancies that account for a high rate of mortality. In 2018, global statistics revealed that approximately over two million new cases of lung cancer were diagnosed [1]. Histologically, lung cancer is classified into two types: non-small cell lung cancer (NSCLC) and small cell lung cancer (SCLC) [2]. Internal factors such as reactive oxygen species (ROS), methylating agents, hydrolytic deamination, and lipid peroxidation-derived aldehydes, as well as external factors such as ultraviolet (UV) light, ionizing radiation, chemicals, and toxins, are in constant interaction with the human genome [3]. The genotoxic stress resulting from various cellular processes (e.g., cellular replication and transcription) is also considered as an endogenous damaging agent [4]. It is well known that radiotherapy plays a crucial role in the treatment of advanced and inoperable NSCLC [5]. However, radio-resistance is a serious challenge that started to limit radiotherapy’s clinical benefits. Researchers have extensively studied the various mechanisms that may have the potential to cause radiotherapeutic resistance [6]. However, tumor heterogeneity along with other factors limited the exact identification of the mechanisms causing radiotherapeutic resistance [7]. Despite the discovery of new promising targeted therapies, there is not yet an effective treatment due to the overall low survival percentage that is to date reported as 17% [8]. Surgery, followed by adjuvant chemo and/or radiotherapy (RT), is currently the most effective treatment option; however, this treatment can only be given to patients with no metastasis [9]. Therefore, as the majority of lung cancer patients are metastatic, the first-line therapeutic option may include chemotherapy, radiofrequency ablation (RFA), RT, immunotherapy, or a combined treatment depending on the disease’s stage and the presence of resistance toward any of the treatment options [10]. In general, lung adenocarcinoma is characterized by recurrent aberrations in several crucial pathways, which activates the RTK/RAS/RAF signaling pathway and the phosphoinositide 3 kinase (PI3K)-mTOR intracellular pathway; alters the tumor protein p53, cell cycle regulators, and oxidative stress; in addition to altering mutations in various chromatin and RNA splicing factors [11].

In every cell cycle, damage to the genetic material (DNA) occurs, and each cell has its own repair machinery to deal with the damage [12]. Cancer cells are comparatively more susceptible to DNA damage, and due to the genomic instability and the acquired changes in cancer cells, they are usually rendered unable to efficiently repair this damage [13]. In the last decade, researchers have identified several new drug targets by using a translational examination of pre-clinical models as well as patient tissues [14,15]. Several drugs that target the proteins involved in DNA damage response (DDR) such as ataxia telangiectasia mutated and Rad3-related protein (ATR), DNA-dependent protein kinase (DNA-PK), and poly-ADP-ribose polymerase (PARP) have been developed [16]. Studies have revealed that DDR targets had a high expression in SCLC, and the data demonstrated that the pre-clinical activity of inhibitors targeting the DDR in SCLC models has shown an instant translational consequence. Many DDR inhibitors such as PARP inhibitors have been developed and gained approval for treating other cancer types [17,18,19,20,21,22]. To date, a limited number of DDR inhibition trials have addressed SCLC patients; however, the data currently available suggest good activity in a group of SCLC cases. In addition, novel biomarkers have started to emerge which may assist in the identification of SCLC subsets that are greatly vulnerable to specific DDR inhibitors [23]. Compared to decades ago, the first-line therapeutic choices remain generally unchanged, and effective therapeutic options in the cases of a disease’s recurrency are also limited, necessitating the discovery and development of innovative strategies for cancer treatment as well as the discovery of confirmed biomarkers [24,25]. Nevertheless, there is new hope for identifying novel therapeutic targets for NSCLC and SCLC patients following the application of emerging next-generation sequencing techniques [25,26]. This review article sheds light on the different DNA repair pathways and some of the inhibitors targeting the proteins involved in DDR such as ATM and ATR, DNA-PK, and PARP. In addition, the current status of DDR inhibitors in clinical settings and future perspectives will be discussed.

## 2. DNA Repair Pathways for the Targeting of Lung Cancer

Recently, researchers have investigated new therapeutic opportunities to target different DDR pathways in aggressive tumors, such as SCLC [27]. To date, the main DNA repair pathways documented are the homologous recombination (HR) repair and non-homologous end-joining (NHEJ) to repair DSBs, base excision repair (BER) to repair SSBs, mismatch repair (MMR) for replication errors repair, and finally the nucleotide excision repair (NER) for bulky adducts repair [21,28,29]. This variety in the DDR pathways allows many of them to work as compensatory mechanisms when the others are compromised [23]. ATM-mediated HR repair and DNA-PK-mediated NHEJ are considered the dominant pathways in the DDR machinery to repair DSBs, which is among the most harmful types of DNA damage [30]. Generally, the standard therapies for the treatment of SCLC are categorized into DNA-damaging agents, causing induction of covalent DNA crosslinks and adducts, such as cisplatin, temozolomide (TMZ), and carboplatin, or SSBs or DSBs, such as ionizing radiation, topotecan, etoposide, and irinotecan [31]. Despite the low survival rates of SCLC, it is notable to be remarkably responsive to treatments that combine multiple DNA-damaging agents. This high sensitivity toward DNA-damaging agents may be due to the underlying genetics that drives the oncogenesis of SCLC [32]. Almost all of the SCLC cases have either homozygous loss or inactivated RB1, which is responsible for the regulation of the G1-S cell cycle checkpoint, and tumor protein 53 (TP53), which is crucial for multiple DDR pathways [33]. This remarkable sensitivity of SCLC to DNA damage proposes an attractive strategy for inhibiting the DNA repair pathways involved in SCLC, and can significantly enhance the efficacy of the currently available therapies [34].

### 2.1. Homologous Recombination (HR) Pathway

**Figure 1:**
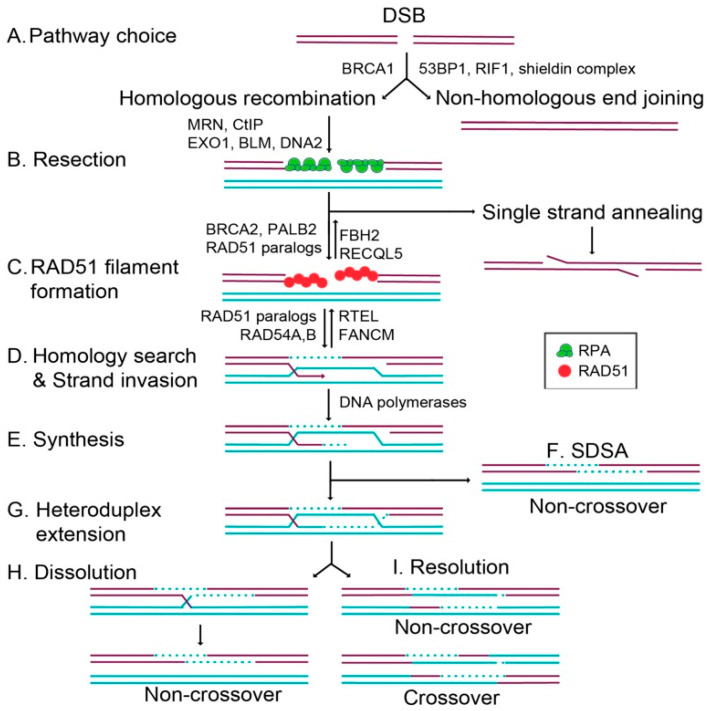
Schematic representation of double-strand break repair by homologous recombination (HR). The scheme explains the mechanism through which cells repair the damage resulting from the formation of a DSB that is displayed in purple lines. As shown in the first step, cells either repair the damage through HR or NHEJ pathways. (A) This choice of any of the two pathways is usually mediated by different factors; the BRCA1 promotes the HR pathway whereas 53BP1, RIF1, and the shieldin complex promote NHEJ. (B) In the second step, a resection by the MRN (MRE11–RAD51–NBS1) complex, CtIP, EXO1, BLM, as well as DNA2 creates 3′ ssDNA overhangs, coated by the trimeric replication protein A (RPA) which is displayed as a complex of green circles. During canonical homologous recombination, RPA undergoes displacement by RAD51 which is displayed as a complex of red circles. An alternative approach is also displayed where RAD51-independent repair may occur through single-strand annealing, which ends up with both the DNA ends ligated together. (C) In the third step, regulation of the RAD51 filament formation by BRCA2, PALB2, and the RAD51 paralogs takes place. Simultaneously, RAD51 is negatively regulated by FBH2 and RECQL5. (D) In the fourth step, RAD51-mediated homology search and strand invasion occurs, which is regulated by the RAD51 paralogs and RAD54A,B. At the same time, RAD51-mediated D loops are negatively regulated by RTEL and FANCM. (E) In the fifth step, the missing information from the homologous template is copied by DNA polymerases as displayed in turquoise. (F) In this step, the D loop undergoes displacement, and the DNA is resolved into a non-crossover product during synthesis-dependent strand annealing (SDSA). (G) Dissolution and resolution of the DNA intermediates may occur if there is a heteroduplex extension and a double Holliday junction formed by second-end capture. (H) Non-crossover products result from dissolution, (I) and both crossover and non-crossover products result from resolution [36].

Homologous recombination is a process through which the DNA is correctly replicated and ensures that detrimental mutation-induced damage does not occur [35]. The HR pathway has been reported to play a crucial role in developing chemotherapeutic resistance in lung adenocarcinoma. This pathway has also been reported to serve a crucial role in the DSBs repair along with NHEJ. Figure 1 represents the HR-mediated DSBs repair [36].

Since the HR pathway applies an error-free repair depending on the DNA homologous strand, it is activated during the cell cycle stages, G0/G1, G1, G2, and S. Sister chromatids at the G2 and S stages are used as a template by this pathway. Moreover, DNA lesions occurring at the replication forks in response to anticancer agents are also considered substrates of the HR repair pathway [37]. For instance, cisplatin and PARP inhibitors have been reported to be more effective on tumors with a defective HR pathway [10]. Many pieces of evidence have been shown to support a distinctive role for ATM in HR, and many HR factors such as BLM gene, breast cancer gene 1 (BRCA1), Nijmegen breakage syndrome 1 (NBS1), carboxy-terminal interacting protein (CtIP), and meiotic recombination 11 (MRE11), are labeled as ATM substrates [38]. It has been reported that cells that carry homozygous ATM kinase-dead mutations show reduced HR expression, and as a consequence, are more sensitive to PARP inhibition, topotecan, and mitomycin C [39]. Homologous recombination deficiency (HRD) is considered common in many cancers and has been shown to lead to an impairment in DNA repair, and hence causes malignancy [40]. Recent studies have investigated HRD in different forms of lung cancers. Diossy et al. have identified a group of lung adenocarcinomas associated with HRD, without losing the key homologous recombination genes BRCA1 and BRCA2. Results have shown that some HR-deficient cancers have an enhanced response to PARP inhibitors as well as to platinum-based chemotherapies; thus it has been concluded that HRD can be considered a biomarker for the responses of these drugs [41]. In other studies, mutations in the BRCA1/2 genes were detected in 5–10% of NSCLC cases [42], which harbor multiple DNA damage checkpoint genes mutations [43]. However, it is still unknown how often these mutations can lead to the HR pathway inactivation in lung cancer. Thus, in order to assess that, an analysis of DNA profiles extracted from the next-generation sequencing data needs to be generated for NSCLC cases [44]. In a recent study, various mutational signatures associated with HRD were derived from whole-genome and whole-exome sequencing data in lung squamous carcinoma and lung adenocarcinoma cases from the cancer genome atlas (TCGA). The results revealed that a group of the cases examined, with and without the presence of biallelic loss of either BRCA1 or BRCA2, showed good signs of HRD. Moreover, HRD-associated mutational signatures have also shown elevated sensitivity to PARP inhibitors in lung cancer cell lines. As a consequence, PARP inhibition therapy may be beneficial in HRD-associated lung cancer cases [45]. In a recent study, it has been demonstrated that a reduced expression of BRCA1 may be a predictor of a better outcome in patients with lung cancer [46]. Thus, depending on the value of BRCA1 expression predicted, the low expression of BRCA1 has been used as a biomarker in NSCLC patients in a Trabectedin clinical trial [47]. However, it was shown later that a low expression of BRCA1 or excision repair cross complementation group 1 (ERCC1) was remarkably associated with good outcomes [48]. On the other hand, the data concerning how mutations or expression of BRCA2 affect the treatment outcomes of patients with lung cancer is still unknown. In summary, more detailed mechanistic studies to investigate these genes in lung cancer are still required in order to involve them in targeted therapies [41]. In a study conducted recently, mutation data in over 1900 samples were analyzed in NSCLC and SCLC cells, and studies proved the HR genes to be highly mutated in both NSCLC and SCLC cancer patients. Results have shown an alteration in the HR genes in 30% of the total lung cancers. These data have suggested that HR genes are often mutated in lung cancer, and targeting lung cancers with HRD is relevant in clinical settings [45]. Nevertheless, HR-associated gene expression has also been linked to the response to RT. For example, an elevated RAD51, NBS, and XRCC3 expression have been shown to cause radio-resistance, whereas reduced expression of XRCC2 causes radiosensitivity [49]. Furthermore, recent studies have investigated the genetic polymorphisms that lead to HRD, and have shown that in response to IR, the single nucleotide polymorphisms in RAD51 and XRCC2 can be considered prognostic factors for the overall survival in NSCLC [50]. A recent study has reported that mutations in BRCA1/2 genes occur in ~2.1% of advanced NSCLC patients [51]. Additionally, in an analysis where more than 100 HR genes were tested, it has been shown that approximately 5% of the total NSCLC cases cause biallelic alterations in an identified HR target gene [52]. In conclusion, inhibiting the HR target is considered promising for identifying most of the HR-proficient NSCLC cases, and hence may enhance the RT beneficial effects [53].

### 2.2. Non-Homologous End Joining (NHEJ) Pathway

**Figure 2:**
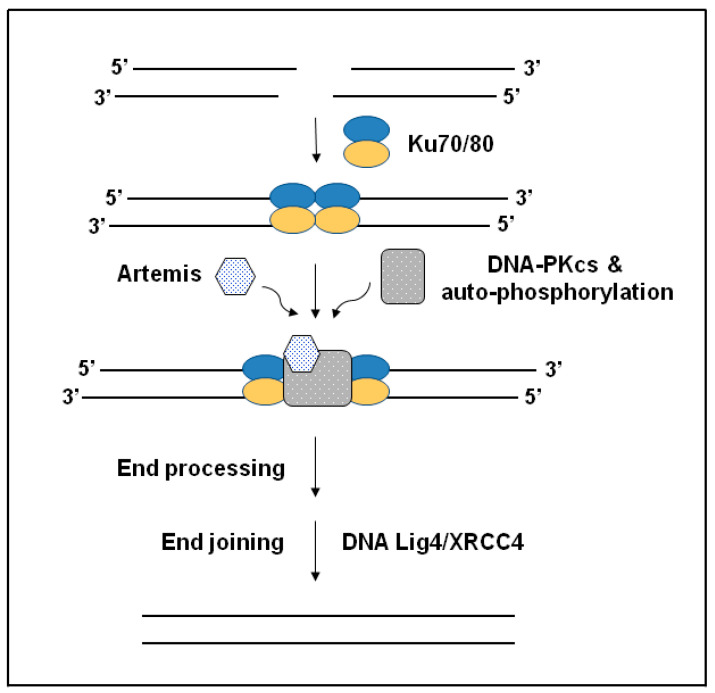
Schematic representation of nonhomologous end-joining (NHEJ) repair pathway in mammalian cells. The scheme shows that following a DSB, Ku which has a high affinity to DNA ends binds to the DNA. This binding causes conformational changes allowing the DNA-PKcs to bind. Ku can also act as an alignment factor for NHEJ accuracy. Upon the formation of the DNA-PK assembly on DNA breaks, this complex causes activation of the serine/threonine protein kinase, and target substrates such as Artemis get phosphorylated, colocalizing at the ends of the broken DNA before end-processing and end-joining events [55].

NHEJ is one of the pathways involved in the DNA DSBs-repairing mechanism. NHEJ can efficiently have a role in all cell cycle phases and allows tumor cells to become resistant to chemotherapeutic drugs [54]. This pathway has five fundamental components, as shown in Figure 2 [55]. In several studies, it has been demonstrated that inhibiting the NHEJ pathway leads to a significant reduction in the resistance against chemotherapeutic drugs. These studies have also acknowledged DNA-PKcs to be the primary target for blocking the NHEJ pathway’s activity due to its central role, as shown in Figure 2, which represents the DSBs repair pathway of the NHEJ [54,56,57,58].

In support of these findings, other studies have reported that higher DNA-PKcs protein has been highly expressed in patients with NSCLC, including adenocarcinoma. Particularly, mutations occurring in Ku80, which is a protein essential to NHEJ repair of DNA DSBs, as shown in Figure 2, have been considered a risk factor in the development of COPD in both humans and mice models [58]. Low levels of Ku80 have been observed in smokers who suffer from COPD, showing high levels of oxidative DNA damage (8-OHdG), which indicates a potential connection between exposure to cigarette smoke and low levels of DNA repair protein expression [59]. Mouse models that lack the Ku70 (XRCC6), which is an essential NHEJ protein, have been shown to develop both the structural and functional changes of COPD with age, and this has been shown to be associated with elevated apoptosis [60]. AKT1 is the main substrate of PI3K, which is responsible for stimulating the IR-induced DSB repair via DNA-PKcs-dependent NHEJ and Rad51-dependent HR. Studies have shown that nuclear localization as well as activation of AKT1 is mandatory for stimulating DSB repair. Therefore, AKT1 is required to induce an immediate expression and activation in the nucleus following IR to recruit DSB repair [61]. Recently, a study was conducted to investigate the subcellular distribution of AKT1 in NSCLC cell lines following IR and stimulation using HER ligands. The data indicated that AKT1 nuclear translocation is a slow process and is dependent on the activity of AKT. These results suggest that both the ionizing radiation (IR) and stimulation with HER family ligands cannot induce the nuclear translocation of AKT1. According to the key role that AKT1 plays in DSB repair, Patritumab, which is a HER3-neutralizing antibody, and the HER3-siRNA have been shown to reduce DSB repair in vitro [62]. Moreover, DDR studies have concluded that following IR or exposure to chemicals, human and mouse lung basal stem cells (BSCs) have a more efficient DNA repair using the NHEJ pathway when compared to alveolar progenitor cells, which has led to cell survival and proliferation [63]. Bioinformatic analysis has shown that lung squamous cell carcinomas (SqCCs) carry a transcriptional fingerprint of human lung BSCs, which suggests that BSCs may act as the original cells of this lung cancer subtype. In conclusion, data have shown that DNA repair that is susceptible to errors is considered a distinctive sign of lung SqCC and suggested that developing drugs that target the NHEJ pathway may prevent and/or treat lung SqCC [63].

### 2.3. Base Excision Repair (BER) Pathway

**Figure 3:**
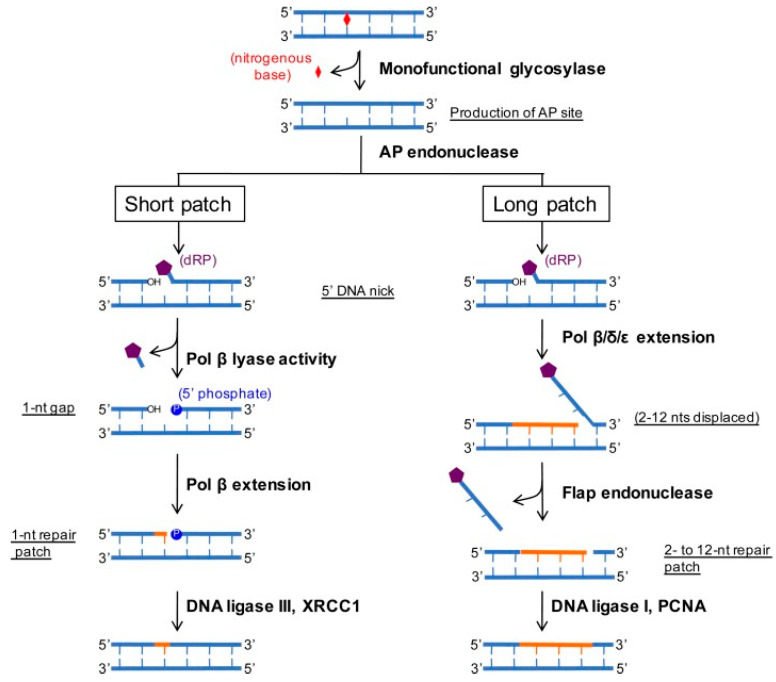
A schematic illustration of the short patch and long patch base excision repair (BER) pathways in eukaryotic cells. A non-helix distorting lesion is recognized by a monofunctional glycosylase that cleaves at the *N*-glucosidic bond, which releases the base. This creates an abasic site that is recognized by an AP endonuclease that creates a 5′ nick. This substrate is then processed by SP or LP repair. SP repair uses the lyase activity of pol β to remove the dRP moiety. Pol β extends 1 nt followed by DNA ligation by the DNA ligase III/XRCC1 complex. LP repair proceeds by pol β, δ, ε to extend ≥2 nts. The displaced DNA is cleaved by flap endonuclease followed by DNA ligation via the coordinated efforts of DNA ligase I and PCNA [64].

The BER mechanism is a DDR pathway that has also been shown to play a role in the chemotherapeutic drug resistance in lung adenocarcinoma. Figure 3 represents the base excision repair (BER) mechanism in mammalian cells [64].

The targeted damages of the DNA by this pathway include oxidative damages, depurination, alkylation as well as deamination, which are all necessary for the growth and development of mammalian cells [65]. When the BER pathways lose their function, severe diseases such as neurological disorders and cancers result [66]. Recent studies have demonstrated that inhibiting the BER pathway causes a significant reduction in chemotherapeutic drug resistance in various cancers, including lung cancer [67]. In NSCLC, a significant correlation between the chemotherapeutic drug resistance and the over-expression of XRCC1 protein has been reported [68]. Besides, EGFR tyrosine kinase inhibitors (EGFR-TKIs) are among the chemotherapeutics that are commonly used for the treatment of patients with advanced NSCLC adenocarcinoma [69]. Among the different mechanisms that have been suggested for the resistance against the EGFRTKIs is PI3K/AKT/mTOR pathway dysregulation [70]. EGFR-TKIs have been shown to exert their action by inhibiting the phosphorylation of Hsp70 and stimulating its ubiquitination in lung adenocarcinoma cells resulting in this protein’s degradation. Hsp70 is essential in the BER pathway since it activates essential enzymes involved in this pathway, which are the APE1 and Pol β enzymes [10]. However, a study conducted recently showed that giving a low dose of erlotinib results in an EGFR T790M mutation on exon 20, which, as a consequence, causes resistance against EGFRTKIs in patients with lung adenocarcinoma. Thus, inactivating the BER pathway by deregulating the Hsp70 is considered critical to forming EGGR T790M mutation in lung adenocarcinoma cells [71]. Afatinib and sirolimus are drugs that are widely used for the treatment of NSCLC. These two drugs were given in combination in a study conducted by Dr. Rosell and colleagues to evaluate their efficacy in reversing acquired EGFR-TKIs resistance. The results have not been promising, noting that further clinical development is required [72]. Dacomitinib is an EGFR-TKIs inhibitor that has also been commonly used to treat patients with metastatic NSCLC [73]. Nevertheless, the BER pathway has also been shown to promote the survival and proliferation of lung cancer cells against organophosphate pesticides (OPP)-induced oxidative stress. In a recent study, it was reported that organophosphate pesticides (OPPs) can initiate oxidative damage in the DNA in an A549 lung adenocarcinoma model [64]. In conclusion, the role of the BER pathway in the development of resistance against chemotherapeutics for lung adenocarcinoma tumors has been illustrated in recent studies [66,67].

### 2.4. Nucleotide Excision Repair (NER) Pathway

**Figure 4:**
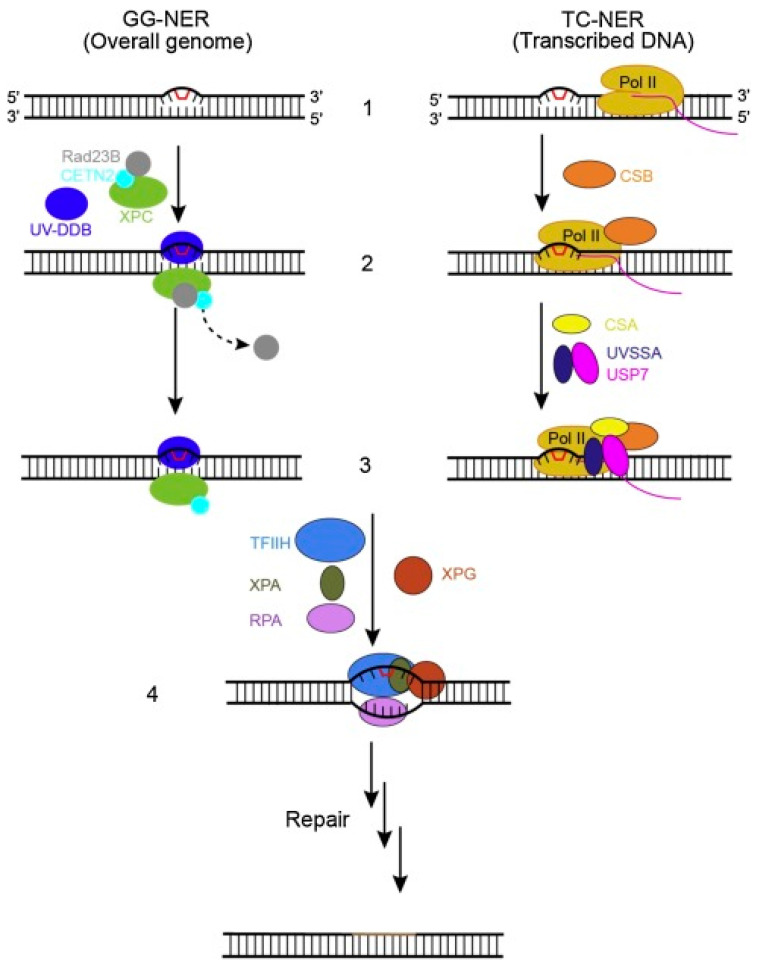
Schematic representation of the two sub-pathways of nucleotide excision repair (NER). In the GG-NER sub-pathway, the damage sensor XPC and the UV excision repair protein homolog B (RAD23B) and centrin 2 (CETN2) complex bind the non-damaged strand with the help of the UV–DDB complex, as shown in the second step. The binding of this complex to the damaged site dissociates the RAD23B from the complex, as displayed in the third step. In the TC-NER, the initiation of damage recognition is performed by the stalling of RNA polymerase II (Pol II). This stalled Pol II activates CSB, resulting in the formation of the Pol II–CSB complex, which serves as a platform for further recruitment of other repairing factors such as CSA and UVSSA-USP7. After recognizing the damage in the first three steps (step 1–3), the TFIIH complex is then recruited to the lesion in both the sub-pathways, along with XPA, RPA, and XPG, as shown in the fourth step. Following the verification of the DNA lesion, dual incisions occur resulting in the removal of the damage-containing DNA short fragment, which is then followed by the synthesis of a new DNA fragment, and the completion of the NER reaction by sealing the final nick by DNA ligase [74].

The NER pathway is a major mechanism in the DDR machinery of mammalian cells that targets massive DNA damage extraction (Figure 4) [74]. This DNA damage is usually composed of nitrogenous bases that are sensitive to reactive oxygen species, UV light, electrophilic chemical mutagens, ionizing irradiation, and chemotherapeutic agents [75]. There are two main mechanisms through which this pathway functions depending on the location where the damage occurred. When the damage occurs on the genome’s side that did not undergo active transcription, the global genome NER (GG-NER) mechanism is then recruited to fix the damage. Otherwise, the other mechanism, the transcription-coupled NER (TC-NER), is recruited [76]. It is well documented in the literature that platinum-based agents, such as cisplatin and carboplatin, are among the most important anti-cancer agents used for the treatment of NSCLC adenocarcinoma patients. These drugs exert their anti-proliferative action by causing DNA damage in cancer cells. Several computational and experimental studies have recently reported the involvement of the NER pathway as well as several related genes in the repair processes of DNA damage, in particular the platinum-based damage [77]. In addition, other studies have reported a significant increase in the ERCC1 expression levels for lung adenocarcinoma patients in particular [78]. Cetuximab is an anti-EGFR agent that is commonly used as a first-line treatment combined with cisplatin or docetaxel for the treatment of advanced NSCLC. Cetuximab exerts its action via the inhibition of the proliferation, invasion, and metastasis of lung cancer cells, in addition to stimulating apoptosis, which leads to high survival rates of NSCLC patients [79]. In a recent study carried out by Li and co-workers, it was reported that ERCC1 overexpression inhibited the activation of the EGFR and stimulated resistance in lung adenocarcinoma cells to the combined therapy of cetuximab and cisplatin [74]. In summary, the NER pathway can be considered one of the critical mechanisms in the DNA repair machinery that contributes to the development of chemotherapeutic drug resistance in lung adenocarcinoma patients [10].

## 3. DDR Inhibitors for a Targeted Treatment of Lung Cancer

When genotoxic stress occurs, there are three phosphatidyl inositol 3-kinase-related protein kinases involved, although each one is activated by different types of injury [23]. ATM has been reported to show a response toward DNA-damaging agents, such as IR, which causes DSBs [80]. Both ATM and ATR kinase have been reported to detect alterations occurring in cell replication upon exposure to ultraviolet light (UV) [81]. Similar to the ATM, another signaling pathway, DNA-PK, has also been shown to be recruited in response to DSBs under certain cellular conditions, such as environmental carcinogens, IR exposure, and chemotherapeutic agents, as well as in cells that have shortened telomeres [82]. In the last decade, the discovery and development of promising DDR targeting agents have opened the door for more exciting avenues for the treatment of several cancers, and SCLC in particular [14]. Many recent studies have reported a high genetic expression of different DDR mediators such as CHK1, PARP, ATM, and ATR in patients with SCLC in comparison to normal lung cells and/or NSCLC cells [3,18,26,41,83,84]. In addition, a high-throughput (HT) small molecule screening has identified other DDR proteins, such as CHK1, to be further explored as candidate targets for treating SCLC patients [85]. Consequently, many DNA repair inhibitors have been recently developed and undergone evaluation in preclinical models as well as clinical trials as candidates for the treatment of SCLC. Although the recently developed DDR inhibitors, such as the PARP inhibitor Talazoparib, have shown promising activity as monotherapies when tested on SCLC models as well as on some patients [86], combining these agents with cytotoxic chemotherapies, other DDR targeting agents [6], or with immunotherapy is expected to have greater clinical responses as this may increase the number of patients responding as well as the duration of the response [87].

### 3.1. ATM/ATR Inhibitors

In the presence of DNA damage, both ATM and ATR have the ability to promote the modification of chromatin via inducing H2AX phosphorylation and hence form foci at the break sites. Following the phosphorylation of H2AX, a γ-H2AX is formed, which allows to the recruitment of other proteins required for the repairing mechanism [81]. ATM and ATR have been reported to have roles in the regulation of the Werner syndrome protein (WRN), which is implicated in the stalled replication fork recovery, hence limiting the fork collapse [88]. They have also been reported to act on BRCA-1, which acts as a scaffold facilitating the activation of the downstream substrates by ATM and ATR, which, when recruited, phosphorylate several substrates [89]. The principal downstream effectors of ATM and ATR are two kinases, CHK2 and CHK1, which transmit the signals to other molecules [82]. In the presence of extended ssDNA stretches with a coating of replication protein A (RPA), ATR is activated by interacting with ATR interacting protein (ATRIP). Once ATR is activated, phosphorylation and activation of multiple targets such as CHK1 take place to form the ATR–CHK1 complex. Upon stimulation, this complex repairs the damage by enforcing the halting of the progression of the cell cycle at the G2-M phase [90]. Recent preclinical observations have suggested that ATR inhibitors may show their activity in TP53 and/or ATM-deficient tumor models when compared to other tumor types [32]. On the other hand, ATM has also been reported to have a role in DNA DSB repair beyond its role in the regulation of p53-mediated apoptosis, and this role in repairing DSBs is through the HR pathway in particular, with an unclear role reported in the NHEJ pathway. In ATM-deficient tumors, DSB repair has been shown to mainly depend on the ATR/CHK1 axis [39]. Recent reports have investigated the activity of ATR inhibitors in both in vitro and in vivo models of SCLC [27]. Remarkably, the clinical results of these studies have been considered promising for further research into ATR inhibitors for the treatment of SCLC [23]. A recent study carried out to test the M6620 pre-treatment in DMS114 cells led to an 1.4-fold increase in TOP1-DPCs following treatment with topotecan for two hours. The results suggested a distinctive role of ATR in the clearance of TOP1 DPCs as well as in repairing TOP1-mediated DNA damage [91]. However, it is worth mentioning that neither the ATM inhibitor (KU55933) nor the DNA-PKcs inhibitor (VS984) has potentiated topotecan-generated TOP1-DPCs [91,92]. In another recent study, the ATRi Ceralasertib has been used along with RT on an NSCLC H460 mouse model. Results demonstrated a remarkable delay in tumor growth [93]. Likewise, ATRi Berzosertib has been shown to enhance the radiation effect in NSCLC brain metastasis patient-derived xenografts (PDXs) models. These findings have been shown to support the continuing clinical trials of Berzosertib combined with whole brain irradiation in NSCLC brain metastasis patients [27]. Nonetheless, Berzosertib has also been shown to enhance the in vivo tumor response to irinotecan, and no additional toxicity has been observed. These findings have provided a foundation for combining TOP1 inhibitor and ATRi in further clinical trials. Similarly, the results of phase I trials where Berzosertib has been combined with topotecan have revealed that this combination has been tolerable and most effective in platinum-refractory SCLC, which in other studies has been reported to have no response to topotecan alone [94]. Moreover, the ATRi Gartisertib has been reported to be significantly synergized with both topotecan and irinotecan in tumor XPD and organoid models derived from humans [95]. A combined therapy of TOP2 inhibitor etoposide and Ceralasertib is in Phase II trial as a candidate treatment for extensive stage SCLC [96]. In a study carried out in 2020 by Byers et al., a combination of AXL inhibitor and ATRi had a significant effect as it decreased the cell proliferation of NSCLC and large cell neuroendocrine carcinoma (LCNEC) cells [97]. As tabulated in Table 1, there are currently various clinical trials of ATM/ATR inhibitors as monotherapies or in combination with other chemotherapeutic drugs for the treatment of SCLS and NSCLC (https://clinicaltrials.gov/, accessed on 20 September 2022).

### 3.2. DNA-PK Inhibitors

As mentioned earlier, chemotherapeutic drugs such as etoposide and doxorubicin and radiotherapy cause DNA damage to exert their anticancer effect [98]. DNA-PK has a crucial role in DSB repair and thus is considered a promising target to treat various cancer types [99]. Many DNA-PK inhibitors with high potency have been recently developed, such as M3814 (Nedisertib), VX-984, and NU7427 [100]. Among all DNA-PK inhibitors, M3814 is an inhibitor with high potency and high selectivity that has been reported to have high activity in preclinical models [101]. Recent studies carried out on lung cancer xenograft models have reported a promising activity of M3814 combined with etoposide and cisplatin for the treatment of lung cancer [102]. Other studies have focused on the investigation of the PI3K-Akt-mTOR signaling cascade, and results have found that this signaling cascade has an essential role in NSCLC tumorigenesis, development, and progression [103,104]. The activation of PI3K-Akt-mTOR has been shown to participate in vital hallmarks of NSCLC, such as sustained cancer growth, resistance to apoptosis, cancer invasion, angiogenesis, metastasis as well as insensitivity to therapies. Therefore, this signaling cascade signifies the key therapeutic target for NSCLC [105,106,107]. The activation of the DNA-PK is seen to promote the repair of DNA damage and causes resistance to cell death by anti-cancer agents. CC-115 is an mTOR kinase blocker with high potency that has been recently discovered and shown to act by inhibiting the activation of mTORC1 and mTORC2. Recent preclinical studies have found that the dual inhibition of mTOR and DNA-PK achieved by CC-115 could have substantial activity in solid tumors [108,109,110,111]. In a recent study carried out by Zheng et al., CC-115 was reported to simultaneously block the activation of both the mTOR and DNA-PK as well as inhibiting the growth of renal cell carcinoma [108]. In addition, another study conducted by Burkel et al. reported that the dual inhibition of mTOR and DNA-PK by CC-115 stimulated melanoma cell death in addition to sensitizing radiation-induced anti-melanoma cell activity [109]. Nonetheless, Tsuji et al. recently reported that CC-115 blocked the DDR and inhibited the growth of ATM-deficient cancer cells [111]. A recent study carried out to test the safety and efficacy of using M3814 in synergy with paclitaxel and etoposide for the treatment of NSCLC has shown that targeting mTOR DNA-PK by CC-115 remarkably hindered the growth of NSCLC cells [83]. M3814 has been reported in other studies to potentiate the anti-cancer effect of both paclitaxel and etoposide in A549 and H460 human NSCLC cell lines. Additionally, tumor regression has also been observed in vivo at tolerated doses. M3814 chemotherapy combination has been shown to induce P53-dependent accelerated senescence of NSCLC cells, which has indicated a possible explanation for the anticancer effect of this therapeutic synergy [98,104]. A recent study has provided a theoretical basis for the use of M3814 and paclitaxel and etoposide combination clinically for optimized NSCLC treatment [112]. A lung cancer model that showed resistance towards Osimertinib has been established and shown to harbor the mutations in EGFR L858R and T790M. Results have revealed that the DDR has been compromised in cells that were resistant to Osimertinib, which indicates that DNA-PK is a key kinase that mediates NHEJ repair and enhances the cells’ sensitivity to Osimertinib. These findings have also revealed an innovative molecular mechanism of Osimertinib resistance and suggested a rationale for combining different DNA-PK inhibitors for the treatment of NSCLC to overcome this acquired Osimertinib resistance [30]. A recent retrospective study on the relationship between the expression of PD-1 and PD-L1 which are immune checkpoints, and the DNA-PK, found a remarkable positive correlation in NSCLC patients [113]. In summary, further investigation is still required in order to clarify this correlation’s significance and its effect on immunotherapy’s effectiveness.

### 3.3. PARP Inhibitors

PARP inhibitors are a class of DDR inhibitors that hinder the repair of DNA and are reported to have anticancer effects [29]. In 2009, the first human clinical trial that used Olaparib as a PARP inhibitor documented the synthetic lethality interaction between this class of inhibitors and the mutations occurring in BRCA1/BRCA2 [114]. Recent studies have investigated the effect of PARP inhibitors in SCLC as it has been reported as sensitive to this class of inhibitors, which has led to further research to test PARP inhibitors as possible therapeutics for treating SCLC [13]. Typically, PARP inhibitors are mostly associated with BRCA1 or BRCA2 genetic mutations and are thought promising for the treatment of lung cancers; however, many other studies revealed that the application of PARP inhibitors is also anticipated to expand to other HR-deficient tumors, thus the discovery of novel biomarkers related to HR abnormalities is considered necessary [115]. Immune checkpoint blockade therapy is promising as a third-line therapeutic option, and its use in first-line therapy has recently been reported with a promising survival benefit, which makes it the new standard of care [115,116]. Typically, many hurdles stand in the way of treating SCLC as this cancer’s biology presents many challenges that limit further therapeutic advances [117]. Additionally, drug resistance has recently begun to emerge; however, the mechanism of resistance is still unclear and is yet to be revealed [118]. As mentioned in previous sections, the SCLC type of lung cancer is considered to have a higher sensitivity to DNA-damaging chemotherapy as compared to NSCLC, accounting for much higher response rates [119]. Resonating this difference, a recent study carried out by Thomas and colleagues under which genomic sequencing of SCLC types was performed, found that the inactivation of RB1 and TP53 DDR regulators was extensive in SCLC [120].

Initially, PARP is considered a novel therapeutic target for the treatment of SCLC following a study carried out by Rudin et al. in which reverse phase protein array (RPPA) analysis of thirty-four SCLC and seventy-four NSCLC cell lines was conducted. The results of this proteomic analysis reported that in the case of SCLC cell lines, the PARP1 mean levels were 2.06-fold higher than NSCLC cell lines (*p* < 0.0001) [34]. In a study carried out by Owonikoko and colleagues, it was demonstrated that veliparib has low efficacy as a monotherapy in vitro when tested on a panel of a total of nine SCLC cell lines in preclinical settings. Unpredictably, veliparib was reported to enhance the cytotoxicity of carboplatin, cisplatin, and etoposide in SCLC cell lines. These data demonstrated that combining these drugs could promote higher inhibition of the growth of the tumor in SCLC PDX [121]. Similarly, another study recently demonstrated that a combination therapy of Olaparib (AZD2281) with cisplatin or etoposide could result in the death of the H82 and H69 cell lines in vitro [20]. In another study carried out by Rudin et al., using SCLC cell lines and patient-derived xenograft (PDX) to test Talazoparib’s efficacy, showed an in vitro synergistic effect with TMZ, with a high combinatorial efficacy degree in vivo [34]. Furthermore, two recent studies have demonstrated that combining either Veliparib or Olaparib with TMZ accounts for more promising results both in vivo and in vitro as compared to monotherapy [119,122]. A recent study carried out by Rudin et al. studied the effect of PARP inhibitors and RT sensitization on SCLC cells. This study investigated the efficacy and potency of a combination therapy Veliparib/Talazoparib and radiotherapy, and showed that Talazoparib exerted a more additive effects in vitro when given in a combination with radiotherapy than with veliparib. However, an in vivo experiment showed that Talazoparib has the ability to enhance the inhibitory effect of RT against the growth of tumor in SCLC PDXs that are resistant to chemotherapy [123]. Nonetheless, an ongoing randomized phase 1/2 study where a combination of carboplatin/etoposide with or without a high dose of veliparib found that cisplatin-etoposide with or without veliparib trial suggested a need for a biomarker selection strategy for the identification of patients who are more likely to benefit from this combination [124]. Table 2 summarizes the current clinical trials of PARP inhibitors as monotherapies or in combination with other chemotherapeutic drugs for the treatment of SCLS and NSCLC (https://clinicaltrials.gov/, accessed on 20 September 2022).

## 4. The Current Status and Future Perspectives

Investigating the genomics of SCLC and discovering new biomarkers is central for providing enhanced and more targeted treatment options [33]. Therefore, whole exome sequencing is believed to assist in the identification of these new targets and biomarkers. For instance, losing the TP53 and RB1 in SCLC has been shown to occur most frequently, thus resulting in replication and proliferation stress as well as early metastasis and a rapid development of chemotherapy resistance [125]. The clinical relevance of finding biomarkers for SCLC is dependent on the preferential targeting of multiple routes, drug combinations, or combining different treatment modalities. Recently, many preclinical studies have acknowledged predictive biomarkers that are responsive to DDR-targeted therapies in SCLC and NSCLC. Several proteins that are involved in this repairing machinery, including PARP, WEE1, ATM and ATR, and checkpoint kinase 1 (CHK1), were all considered attractive targets for the treatment of SCLC and NSCLC [23]. These promising targets have been shown to prevent the damaged cells or the cells that have an incompletely replicated DNA from entering into mitosis and therefore case suppression of the replication stress, and hence resulting in cell death [29,82,84,91,99,106,126]. Adavosertib (AZD1775) has been reported to have high selectivity and high potency as a WEE1 inhibitor when used as a monotherapy or combined with PARP inhibitors or other chemotherapeutic drugs [21]. Recent genomic sequencing has identified the Schlafen 11 (SLFN11), which is a predictive biomarker to predict the PARP inhibition sensitivity when used as a monotherapy for the treatment of SCLC [34]. The expression of SLFN11 is higher in SCLC and its expression has been shown to decrease remarkably following treatment with veliparib. Moreover, the treatment with TMZ has been reported with beneficial effects in patients with SCLC, particularly in a subgroup where the MGMT promoter methylation is present [127]. On the other hand, new strategies for testing different DDR-inhibitor combinations or targeting multiple pathways are yet to be explored [128]. In addition, the intra-tumor heterogeneity is an ongoing challenge in the targeted treatment of SCLC, and a strategy of blocking multiple routes of growth in order to block the growth of the tumor stands as a promising solution. Recent studies have found that co-targeting DDR proteins (i.e., PARP and CHK1) may increase the PD-L1 expression and enhance the anti-tumor immune response in SCLC [126]. Thus, a strategy where DDR targeting is combined with immunotherapy could also be promising. In summary, with the various biomarkers that have either been recently discovered or are the subject of ongoing investigations, designing future trials is hoped to allow for studying targeted treatments in a biomarker-enriched population, which is defensible for the improvement of prognosis for SCLC patients [129].

## 5. Conclusions

In every cell cycle, damage to the genetic material (DNA) occurs, and each cell has its repairing machinery to deal with the damage [12]. In the case of tumor cells, the DNA damage is comparatively greater than the damage occurring in normal cells due to the use of chemotherapy and/or radiation, and the repairing system of DNA is usually inactive in tumor cells unlike normal cells [13]. Inhibiting a target that promotes the HR pathway is a promising approach to identifying most of the HRD-NSCLC cases, and hence may enhance the beneficial effects of RT [53]. Several drugs that target the proteins involved in DDR, such as ATM and ATR, DNA-PK, and PARP have been developed [16]. To date, a limited number of DDR inhibitor trials have included SCLC patients, although the data currently available points to good activity in a group of patients suffering from SCLC. In addition, novel biomarkers have started to emerge which may assist in the identification of SCLC subsets that are greatly vulnerable to specific DDR inhibitors [23]. Despite the low survival rates of SCLC, it is notable to be remarkably responsive to treatments that combine multiple DNA-damaging agents. This high sensitivity toward DNA-damaging agents may be due to the underlying genetics that drive the oncogenesis of SCLC [32]. In the last decade, the discovery and development of promising DDR targeting agents have opened the door for more exciting avenues for the treatment of several cancers, and SCLC in particular [14]. Although the recently developed DDR inhibitors such as the PARP inhibitor Talazoparib have shown promising activity as monotherapies when tested on SCLC models [86], combining these agents with cytotoxic chemotherapies, other DDR targeting agents [6], or with immunotherapy is expected to have greater clinical responses as this may increase the number of patients responding as well as the duration of the response [87]. Investigating the genomics of SCLC and discovering new biomarkers is central for providing enhanced and more targeted treatment options [33]. New strategies to test different DDR-inhibitors combinations or to target multiple pathways are yet to be explored [128]. With the various biomarkers that have either been recently discovered or are the subject of an ongoing investigation, designing future trials is hoped to allow for studying targeted treatments in a biomarker enriched population, which is defensible for the improvement of prognosis for SCLC patients [129].

## Figures and Tables

**Table 1:** The current clinical trials of ATM/ATR inhibitors as monotherapies or in combination with other chemotherapeutic drugs for the treatment of SCLS and NSCLC.

ID	Condition or Disease	Intervention/Treatment	Status
NCT02487095	Carcinoma, Non-small cell lung Ovarian neoplasms Small cell lung carcinoma Uterine cervical neoplasms carcinoma, Neuro-endocrine Extra-pulmonary Small cell cancer	Drug: Topotecan Drug: VX-970 (M6620)	Active, Not recruiting
NCT02487095	Carcinoma, non-Small cell lung Ovarian neoplasms Small cell lung carcinoma Uterine cervical Neoplasms carcinoma, Neuroendocrine extrapulmonary Small cell cancer	Drug: Topotecan Drug: VX-970 (M6620)	Active, Not recruiting
NCT02589522	Metastatic lung neuroendocrine neoplasm Metastatic lung non-small cell carcinoma Metastatic lung small cell carcinoma Metastatic malignant neoplasm in the brain stage IV lung cancer AJCC v8Stage IVA lung Cancer AJCC v8Stage IVB lung Cancer AJCC v8	Drug: Berzosertib Other: Quality-of-Life Assessment Procedure: Therapeutic Conventional Surgery Radiation: Whole-Brain Radiotherapy	Active, Not recruiting
NCT04768296	Small cell lung cancer	Drug: Berzosertib Drug: Topotecan	Active, Not recruiting
NCT04216316	Lung non-small cell squamous carcinoma Stage IV lung cancer AJCC v8	Drug: Berzosertib Procedure: Biospecimen Collection Drug: Carboplatin Drug: Gemcitabine Hydrochloride Biological: Pembrolizumab	Recruiting
NCT05450692	Advanced or metastatic non-small cell lung cancer	Drug: Ceralasertib Drug: Durvalumab Drug: Docetaxel	Not yet recruiting
NCT04699838	Extensive stage small cell lung cancer	Drug: Cisplatin Drug: Carboplatin Drug: Etoposide Drug: Durvalumab Drug: Ceralasertib	Recruiting
NCT04768296	Small cell lung cancer	Drug: Berzosertib Drug: Topotecan	Active, not recruiting
NCT04216316	Lung non-small cell squamous carcinoma Stage IV lung cancer AJCC v8	Drug: Berzosertib Procedure: Biospecimen Collection Drug: Carboplatin Drug: Gemcitabine Hydrochloride Biological: Pembrolizumab	Recruiting
NCT04826341	HRD cancer SCLC Advanced solid tumors	Drug: Berzosertib Drug: Sacituzumab Govitecan	Recruiting

**Table 2:** The current clinical trials of PARP inhibitors as monotherapies or in combination with other chemotherapeutic drugs for the treatment of SCLS and NSCLC.

ID	Condition/Disease	Intervention	Status
NCT01638546	Recurrent small cell lung carcinoma	Other: Laboratory Biomarker Analysis Other: Placebo Drug: Temozolomide Drug: Veliparib	Completed
NCT04728230	Extensive stage lung small cell carcinoma Stage IV lung cancer AJCC v8 Stage IVA lung cancer AJCC v8 Stage IVB lung cancer AJCC v8	Drug: Carboplatin Biological: Durvalumab Drug: Etoposide Drug: Olaparib Radiation: Radiation Therapy	Recruiting
NCT04701307	Lung small cell carcinoma Neuroendocrine carcinoma Stage III lung cancer AJCC v8 Stage IIIA lung cancer AJCC v8 Stage IIIB lung cancer AJCC v8 Stage IIIC lung cancer AJCC v8	Biological: Dostarlimab Drug: Niraparib	Recruiting
NCT03672773	Recurrent extensive stage small cell lung carcinoma refractory extensive stage small cell lung carcinoma	Drug: Talazoparib Drug: Temozolomide	Active, Not recruiting
NCT02289690	Small cell lung cancer	Drug: Veliparib Drug: Carboplatin Drug: Etoposide Drug: Placebo	Completed
NCT04538378	EGFR-mutated non-small cell lung carcinoma Small cell/neuroendocrine	Drug: Olaparib Drug: Durvalumab	Recruiting
NCT03830918	Advanced malignant solid neoplasm Extensive stage lung small cell carcinoma Stage III lung cancer AJCC v8 Stage IIIA lung cancer AJCC v8 Stage IIIB lung cancer AJCC v8 Stage IIIC lung cancer AJCC v8 Stage IV lung cancer AJCC v8 Stage IVA lung cancer AJCC v8 Stage IVB lung cancer AJCC v8	Biological: Atezolizumab Drug: Niraparib Other: Quality-of-Life Assessment Other: Questionnaire Administration Drug: Temozolomide	Recruiting
NCT01286987	Advanced or recurrent solid tumors Breast neoplasms Ovarian cancer, epithelial Ewing sarcoma Small cell lung carcinoma Prostate cancer Pancreas cancer	Drug: Talazoparib	Completed
NCT03958045	Small cell lung cancer	Combination Product: Rucaparib and Nivolumab	Recruiting
NCT02769962	Urothelial carcinoma Urothelial cancer Lung neoplasms Small cell lung cancer Prostate cancer	Drug: EP0057 Drug: Olaparib	Recruiting
NCT01082549	Squamous cell lung cancer	Drug: gemcitabine/carboplatin Drug: gemcitabine/carboplatin plus Iniparib	Completed
NCT01788332	Non-small cell lung cancer	Drug: Olaparib Other: Placebo	Unknown
NCT01086254	Non-small cell lung cancer stage IV	Drug: Iniparib Drug: gemcitabine Drug: cisplatin	Completed
NCT05392686	Non-small cell lung cancer	Drug: PD-1 inhibitor Drug: PARP inhibitor	Recruiting
NCT04380636	Lung neoplasms Carcinoma, non-small cell lung	Biological: Pembrolizumab Drug: Olaparib Drug: Placebo for Olaparib Drug: Etoposide Drug: Carboplatin Drug: Cisplatin Drug: Paclitaxel Drug: Pemetrexed Radiation: Thoracic Radiotherapy Drug: Durvalumab	Recruiting
NCT01560104	Non-small cell lung cancer	Drug: Veliparib Drug: Carboplatin Drug: Paclitaxel Drug: Placebo	Completed
NCT02944396	Non-small cell lung cancer	Drug: Pemetrexed Drug: Nivolumab Drug: Paclitaxel Drug: Veliparib Drug: Carboplatin	Completed
NCT02264990	Non-squamous non-small cell lung cancer	Drug: Paclitaxel Drug: Carboplatin Drug: Cisplatin Drug: Veliparib Drug: Pemetrexed	Completed
NCT04538378	EGFR-mutated non-small cell lung Carcinoma Small cell/neuroendocrine	Drug: Olaparib Drug: Durvalumab	Recruiting
NCT02106546	Squamous non-small cell lung cancer	Drug: Carboplatin Drug: Veliparib Drug: Paclitaxel Drug: Placebo to veliparib	Completed

## Data Availability

Not applicable.

## Abbreviations

ATM	Ataxia telangiectasia mutated
ATR	Rad3-related protein
ATRIP	ATR interacting protein
BER	Base excision repair
COPD	Chronic obstructive pulmonary disease
DDR	DNA damage response
DNA-PK	DNA-dependent protein kinase
DNAPKcs	DNA-dependent protein kinase catalytic subunit
DSBs	Double-strand breaks
EGFR-TKIs	EGFR tyrosine kinase inhibitors
ERCC	Excision repair cross complementation
GG-NER	Global genome NER
HR	Homologous recombination
HRD	Homologous recombination deficiency
IR	Ionizing radiation
LCNEC	Large cell neuroendocrine carcinoma
MMR	Mismatch repair
NER	Nucleotide excision repair
NHEJ	Non-homologous end joining
NSCLC	Non-small cell lung cancer
OPPs	Organophosphate pesticides
PARP	Poly-ADP-ribose polymerase
PDX	Patient-derived xenograft
RFA	Radiofrequency ablation
ROS	Reactive oxygen species
RPA	Replication protein A
RPPA	Reverse phase protein array
RT	Radiotherapy
SCLC	Small cell lung cancer
SLFN11	Schlafen 11
SqCCs	Squamous cell carcinomas
SSBs	Single-strand breaks
TCGA	The cancer genome atlas
TC-NER	Transcription-coupled NER
TMZ	Temozolomide
UV	Ultraviolet
WRN	Werner syndrome protein
XRCC4	X-ray repair complementing defective repair in Chinese-hamster cells 4
